# Overexpression of *FAM83H-AS1* indicates poor patient survival and knockdown impairs cell proliferation and invasion via MET/EGFR signaling in lung cancer

**DOI:** 10.1038/srep42819

**Published:** 2017-02-15

**Authors:** Jie Zhang, Shumei Feng, Wenmei Su, Shengbin Bai, Lei Xiao, Lihui Wang, Dafydd G. Thomas, Jules Lin, Rishindra M. Reddy, Philip W. Carrott, William R. Lynch, Andrew C. Chang, David G. Beer, You-min Guo, Guoan Chen

**Affiliations:** 1 Xian Jiaotong University, Xi’an, China; 2 Department of Surgery, University of Michigan, Ann Arbor, Michigan, USA; 3 Xinjiang Medical University, Urumqi, China; 4 Guangdong Medical University, Zhanjiang, China; 5 Guangxi Medical University, Nanning, China; 6 Department of Pathology, University of Michigan, USA.

## Abstract

Whole transcriptome analyses of next generation RNA sequencing (RNA-Seq) data from human cancer samples reveled thousands of uncharacterized non-coding RNAs including long non-coding RNA (lncRNA). Recent studies indicated that lncRNAs are emerging as crucial regulators in cancer processes and potentially useful as biomarkers for cancer diagnosis and prognosis. To delineate dysregulated lncRNAs in lung cancer, we analyzed RNA-Seq data from 461 lung adenocarcinomas (LUAD) and 156 normal lung tissues. *FAM83H-AS1*, one of the top dysregulated lncRNAs, was found to be overexpressed in tumors relative to normal lung and significantly associated with worse patient survival in LUAD. We verified this diagnostic/prognostic potential in an independent cohort of LUAD by qRT-PCR. Cell proliferation, migration and invasion were decreased after *FAM83H-AS1* knockdown using siRNAs in lung cancer cells. Flow cytometry analysis indicated the cell cycle was arrested at the G2 phase after *FAM83H-AS1* knockdown. Mechanistically, we found that MET/EGFR signaling was regulated by *FAM83H-AS1*. Our study indicated that *FAM83H-AS1* plays an important role in lung tumor progression and may be potentially used as diagnostic/prognostic marker. Further characterization of this lncRNA may provide a novel therapeutic target impacting MET/EGFR signaling.

Lung cancer is the leading cause of cancer-related deaths globally and is histologically-defined as either small cell lung cancer (SCLC) or non-small cell lung cancer (NSCLC), with the latter accounting for 80% of all lung cancers^1^,^2^. NSCLC is a heterogeneous disease, with the most common subtypes being adenocarcinoma (LUAD) and squamous cell carcinoma (SCC). These subtypes represent distinct clinical entities, typically requiring different treatment options. Among these histological subtypes there exist cancers with diverse clinical outcomes, revealing heterogeneity in disease aggressiveness and underlying molecular alterations^3^,^4^,^5^. Indeed, the poor prognosis associated with lung cancer (15.7–18% 5-year survival) is related to the complex cellular, molecular and tumor microenvironment factors that impart a unique biological basis to an individual’s disease^2^. Discovery of oncogenic driver alterations have helped improve the outcomes in specific subtypes of patients with lung cancer, however the majority of the patients with lung cancer do not have an actionable molecular aberration^6^,^7^. Therefore, there is a vital need for new biomarkers and the identification of alternative treatments.

Long non-coding RNAs (lncRNAs) are RNA transcripts that are greater than 200 bp in length and lack an open reading frame encoding a protein^8^,^9^,^10^,^11^. LncRNA exhibit tissue or cancer specific expression patterns. In lung cancer, antisense RNA, long intergenic non-coding RNA (lincRNA), and processed transcripts are the most frequently expressed lncRNAs^12^. In the past few years, lncRNAs have emerged as novel mechanisms in mediating cancer biology^13^,^14^,^15^,^16^,^17^,^18^, although most lncRNAs remain undiscovered. LncRNAs appear to be involved in tumorigenesis, cell proliferation, differentiation, migration, immune response, apoptosis, and angiogenesis^13^,^16^,^19^,^20^,^21^. Several mechanisms associated with lncRNAs in tumor biological processes are remodeling of chromatin (*HOTAIR, XIST, ANRIL*), transcriptional co-activation or co-repression (*H19, LincRNA-p21*), protein inhibition (*TERRA*), post-transcriptional modifiers (*MALT1*) and decoy elements (*PTENP1*)^22^,^23^,^24^,^25^.

High-throughput RNA sequencing (RNA-Seq) of human cancer has shown remarkable potential to identify both novel markers of disease and uncharacterized aspects of tumor biology, particularly lncRNA species^11^,^12^,^26^,^27^. We recently generated transcriptome data using next-generation RNA-Seq to reveal both protein coding and lncRNA expression patterns in lung cancer^7^,^11^,^12^.

In the present study, we characterized a dysregulated lncRNA in lung cancer, *FAM83H Antisense RNA1 (FAM83H-AS1*), for its diagnostic/prognostic potential and role in cancer progression. We validated its differential expression in an independent LUAD cohort and established its diagnostic/prognostic importance. We next performed *in vitro* studies to delineate its oncogenic roles in cell proliferation, invasion and migration. Finally, we attempted to reveal which cancer related pathway was affected using a *FAM83H-AS1* knockdown assay.

## Results

### *FAM83H-AS1* expression is increased in lung adenocarcinomas and is associated with worse patient survival

In our previous study^12^, we analyzed 3 large RNA-Seq data sets representing independent tumor cohorts. These data sets are the University of Michigan (UM) cohort^7^ including 67 LUADs and 6 matched normal lung tissues, the Korean cohort (Seo)^28^ including 85 LUADs and 77 normal lung samples, and The Cancer Genome Atlas (TCGA) LUAD cohort^29^ including 309 LUADs and 73 normal lung samples. In order to identify lncRNAs whose expression patterns may have significant clinical utility, we performed a Receiver Operating Characteristic (ROC) curve analysis. The area under the curve (AUC) values was used to select the list of top differently expressed lncRNAs in LUAD. There were a total of 182 lncRNAs that had an AUC value greater than 0.7 and 99 lncRNAs that had an AUC value less than 0.25 in all 3 data sets^12^.

**Figure 1:**
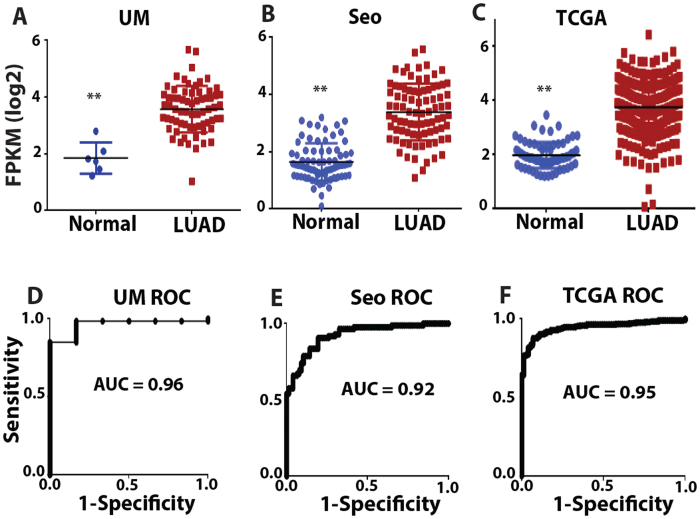
Increased *FAM83H-AS1* expression in lung ADs. (**A–C**) scatterplots of *FAM83H-AS1* expression levels in LUAD and normal lung tissue samples in UM (67 LUAD vs. 6 Normal), Seo (85 LUAD vs. 77 Normal) and TCGA (309 LUAD vs. 73 Normal) RNA-Seq datasets (LUAD vs. Normal, p < 0.001 in all 3 data sets). (**D–F**) Receiver Operating Characteristic (ROC) curves of *FAM83H-AS1* in UM, Seo and TCGA RNA-Seq data sets (LUAD vs. Normal). The area under the curve (AUC) >0.9 in all 3 data sets.

**Figure 2:**
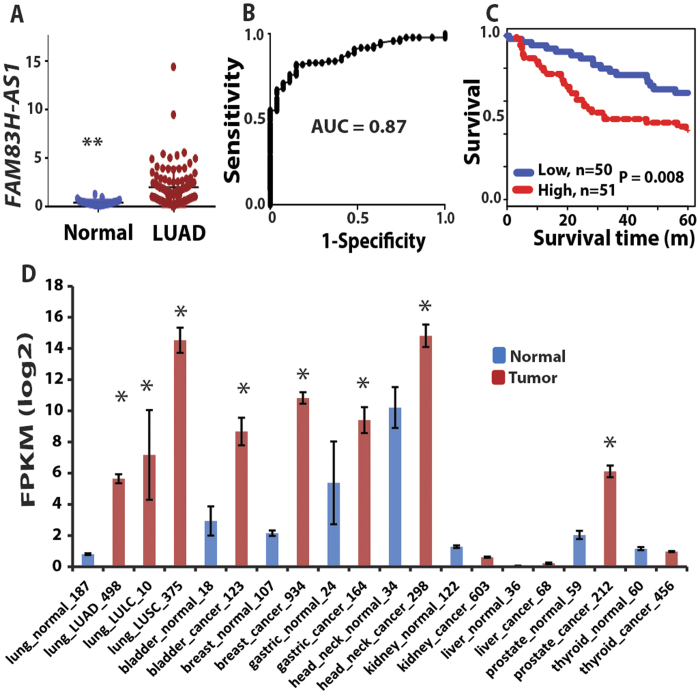
Validation of *FAM83H-AS1* expression in an independent cohort of LUADs by qRT-PCR. (**A–C**) validation of *FAM83H-AS1* expression in an independent set of samples including 19 normal lung tissues and 101 LUAD tissues using qRT-PCR. *FAM83H-AS1* expression was increased in tumors (vs. normal, **p < 0.001) (**A**), ROC curve of classifying the tumors from normal based on *FAM83H-AS1* expression (AUC = 0.87) (**B**), and Kaplan-Meier survival curve indicated higher *FAM83H-AS1* expression was unfavorable for patient survival (log-rank test, p = 0.008) (**C**). (**D**) Higher expression (FRKM log2 value > 2) of *FAM83H-AS1* was also present in squamous cell (LUSC) and large cell lung cancer (LULC), as well as bladder, breast, gastric, head/neck, and prostate cancers, whereas lower expression was present in kidney, liver, and thyroid cancers (FRKM log2 value < 2) based on RNA-Seq expression data from MiTranscriptome. *Tumor vs. normal, p < 0.05.

Among the most dysregulated lncRNAs, *FAM83H-AS1* was found to be significantly increased in LUADs (Fig. 1A–C) and had AUC >0.9 in all 3 cohorts (Fig. 1D–F). Because *FAM83H-AS1* is a novel lncRNA, there are no probe sets present on Affymetrix U133Plus2.0. There are *FAM83H-AS1* probes on the Affymetrix exon array, but we did not find large number of lung tumor samples (or samples having survival information) using this platform on the GEO (Gene Expression Omnibus) database. In order to validate the expression of *FAM83H-AS1* as well as evaluate its prognostic significance in lung cancer, we examined *FAM83H-AS1* expression in an independent cohort of LUAD from UM including 101 lung ADs and 19 normal lung tissues using qRT-PCR. The boxplot shows that *FAM83H-AS1* expression was significantly higher in cancer tissues as compared to normal lung tissues (p < 0.001) (Fig. 2A). The AUC = 0.87 indicates *FAM83H-AS1* expression could significantly separate the tumors from normal lung tissues (Fig. 2B). We also found that *FAM83H-AS1* expression was significantly associated with unfavorable survival in patients with lung cancer (Fig. 2C). We did not find that *FAM83H-AS1* expression levels were associated with tumor stage, differentiation and other clinical variables in this validation set (Supplementary Table 1).

In order to discover the *FAM83H-AS1* expression status in other types of cancer, we examined RNA-Seq data based on 6,220 different types of cancer from one lncRNA database, MiTranscriptome^26^. The higher expression of *FAM83H-AS1* (FRKM log2 value >2) was also found in squamous cell (LUSC) and large cell lung cancer (LULC) as well as bladder, breast, gastric, head/neck, prostate cancers. Lower expression was found in kidney, liver, and thyroid cancers (FRKM log2 value <2) (Fig. 2D). By comparing tumor vs. normal, we found that bladder, breast, gastric, head/neck, and prostate cancers were significantly increased. However, kidney, liver and thyroid cancer were not significantly changed between tumor and normal (Fig. 2D). Taken together, *FAM83H-AS1* was highly expressed in not only lung cancer but also in other types of cancers and could be possibly used as a diagnostic or prognostic marker for various cancers.

### Cell proliferation, invasion and migration were impaired after *FAM83H-AS1* knockdown

**Figure 3:**
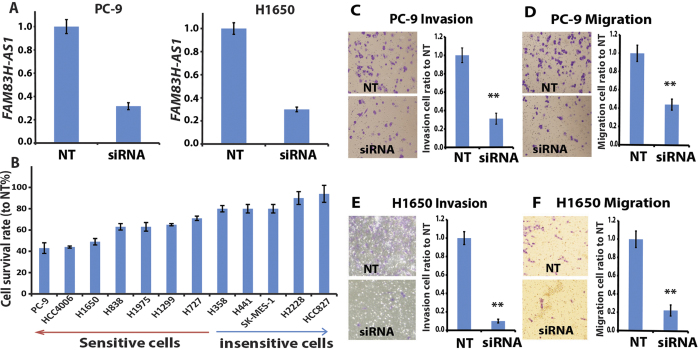
*FAM83H-AS1* involved in tumor cell proliferation and invasion. (**A**) The knockdown efficiency of *FAM83H-AS1* siRNA in PC-9 and H1650 cells measured by qRT-PCR. The *FAM83H-AS1* expression decreased more than 70% after *FAM83H-AS1* siRNA (10 nM SMARTpool, at 48 h) transfection. (**B**) Cell proliferation was altered after *FAM83H-AS1* siRNAs treatment (96–120 h) in 12 lung cancer cell lines. (**C–F**) cell invasion and migration decreased after *FAM83H-AS1* knockdown by siRNA in both PC-9 and H1650 cell lines (20X) analyzed using Boyden chambers assay. siRNA = *FAM83H-AS1* siRNA; NT = non-targeting control siRNA.

Since *FAM83H-AS1* is overexpressed in most cancer tissues, we hypothesize that this lncRNA may play an oncogenic role in lung cancer progression. For this purpose, we examined cell proliferation, invasion and migration status after knockdown of *FAM83H-AS1* by siRNA in lung cancer cells. The knockdown efficiency was 60–70%, measured by qRT-PCR, in 12 lung cancer cell lines (Fig. 3A and Supplementary Figure 1) with different genomic background (Supplementary Table 2) and different *FAM83H-AS1* expression levels examined by RNA-Seq and RT-PCR (Supplementary Figure 2). Next, we tested the cell proliferation status after *FAM83H-AS1* knockdown in these cell lines. We found that 7 out of 12 cell lines showed a significant decrease in cell proliferation (>30%). Surprisingly, we found that the cell growth was reduced in 4/5 of the tested EGFR mutated cells (Fig. 3B and Supplementary Table 2), including EGFR tyrosine kinase inhibitor (TKI) sensitive cells (PC-9) and resistant cells (HCC4006, H1975 and H1650). This result indicated that *FAM83H-AS1* could affect cell proliferation even in EGFR TKI resistant cells. There were 5 cell lines not affected including two *KRAS* mutated cells (H358 and H441) and three *TP53* mutated cells (H441, SKMES-1 and H2228, which also had a *EML4*-*ALK* fusion). HCC827 is an *EGFR* mutated and TKI sensitive cell as well as being *EGFR* amplified. We did not find a correlation between *FAM83H-AS1* gene expression levels and a proliferation effect (Supplementary Figure 3). These results suggest that some cells demonstrate growth independent of *FAM83H-AS1*.

Since cell proliferation was affected in most *EGFR* mutated cells following *FAM83H-AS1* knockdown, we selected the two most affected lung cancer cells, PC-9 (TKI sensitive) and H1650 (TKI resistant), for cell invasion and migration studies using Boyden chamber matrix assays. We found that cell invasion (with matrigel) was reduced by 70% versus control when *FAM83H-AS1* was knocked down in both PC-9 and H1650 cell lines (Fig. 3C,E). Similarly, *FAM83H-AS1* knockdown resulted in more than 60% decrease in cell migration (Fig. 3D,F). Colony formation was also decreased after *FAM83H-AS1* knockdown (data not shown).

We found that *FAM83H-AS1* could affect cell growth on one subset of lung cancer cells *in vitro* (Fig. 3B). Higher expression of this lncRNA is correlated with poor patient survival in clinical samples. To test if *FAM83H-AS1* expression status is correlated with expression of the cell proliferation marker Ki-67 in clinical lung cancer samples, we performed Ki-67 immunohistochemistry (IHC) staining on 97 lung cancer tissues using tissue array. These 97 samples were also used for *FAM83H-AS1* expression by RT-PCR in this study (Fig. 2C). Ki-67 staining was scored as 0 (negative), 1 (<10% positive cells), 2 (10–50% positive cells), or 3 (>50% positive cells) (Supplementary Figure 4). We did not find a significant correlation between *FAM83H-AS1* expression and Ki-67 staining (Supplementary Figure 5).

Taken together, these results indicated that *FAM83H-AS1* may not only be a potential biomarker for diagnosis and prognosis of lung cancer, but also play a critical role in lung cancer progression including cell proliferation, invasion, migration and colony formation.

### *FAM83H-AS1* knockdown causes cell cycle arrest at G2

**Figure 4:**
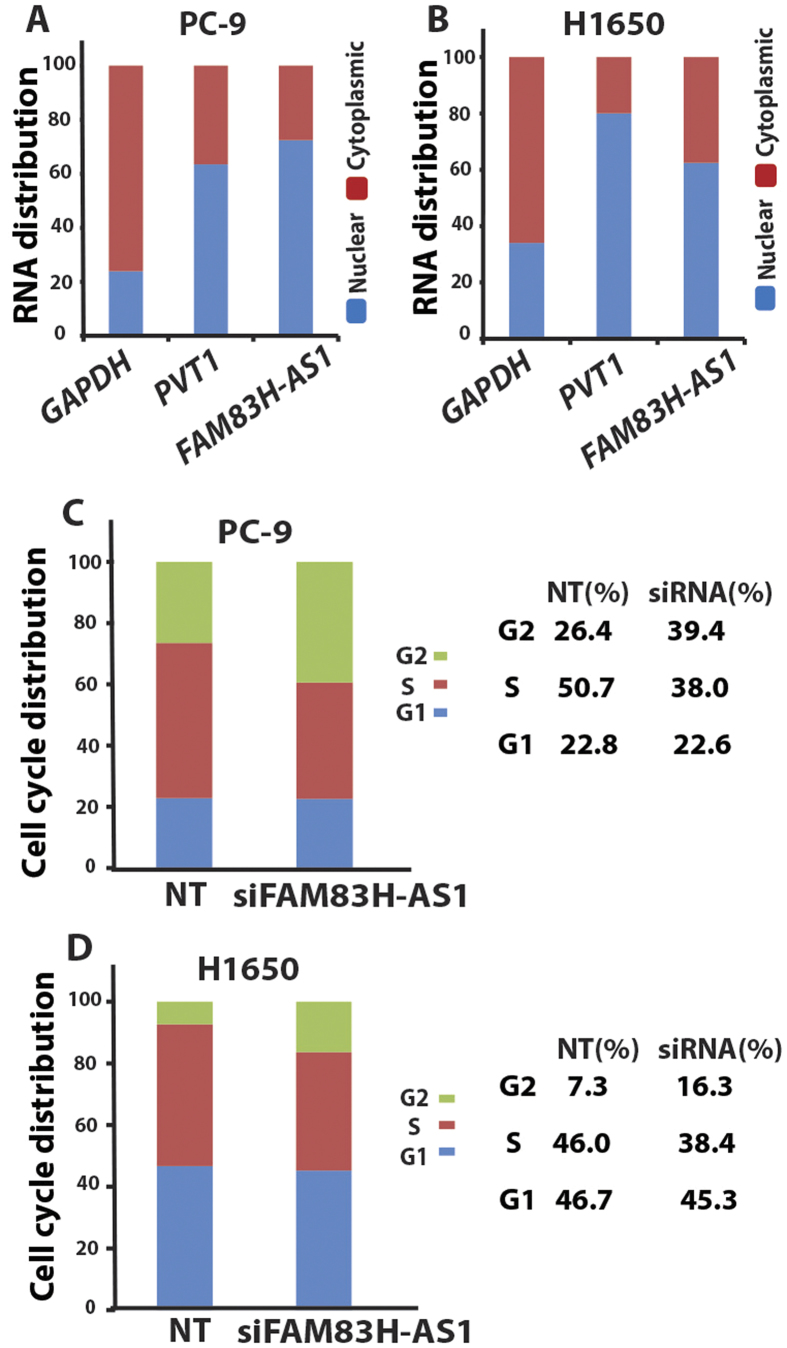
*FAM83H-AS1* cell location and cell cycle regulation. (**A,B**) *FAM83H-AS1* is mainly located at nuclear measured by RT-PCR. GAPDH was used as cytoplasmic control and PVT1 as nuclear control. (**C,D**) *FAM83H-AS1* knockdown using siRNA caused G2 arrested in both PC-9 (G2 cells increased from 26.4% to 39.4% after siRNA treatment) and H1650 (G2 cells increased from 7.3% to 16.3% after siRNA treatment) lung cancer cell lines. Right panel is the actual number of cell cycles calculated from left panel.

We first looked at the cellular localization of *FAM83H-AS1* in PC-9 and H1650 cells. RNA was isolated from total, nuclear and cytoplasmic fractions of these two cell lines. Quantitative RT-PCR indicated *FAM83H-AS1* expression was mainly in the nuclear fraction (Fig. 4A,B). Next, to determine whether *FAM83H-AS1* affects cell cycle regulation, we performed flow cytometry to explore the effect on the cell cycle after *FAM83H-AS1* knockdown. We found that *FAM83H-AS1* knockdown by siRNA induced cell cycle arrest at the G_2_ phase in both PC-9 and H1650 cells (Fig. 4C,D). These results suggest that decreased tumor cell growth by *FAM83H-AS1* knockdown might be through G_2_ arrest.

### *FAM83H-AS1* regulates MET/EGFR signaling

**Figure 5:**
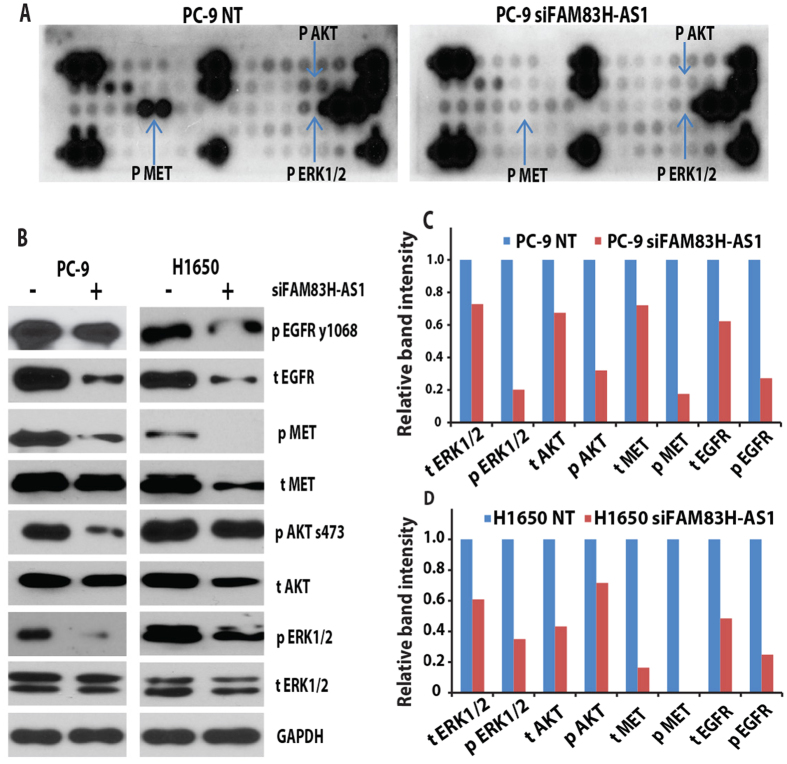
*FAM83H-AS1* regulated MET/EGFR signaling. (**A**) Receptor tyrosine kinase phosphorylation antibody array indicated phosphor-MET is the most significantly decreased protein after *FAM83H-AS1* knockdown by siRNA (10 nM at 96 h) as compared to NT in PC-9 cell. Phosphor-AKT and ERK1/2 were also decreased. (**B**) Western blot showing MET, EGFR, AKT and ERK1/2 were decreased after *FAM83H-AS1* siRNA treatment on PC-9 and H1650 lung cancer cell lines. (**C,D**) Quantitative analysis of image in B using Image J software. GAPDH was used as protein loading control (NT = 1). Abbreviation: for protein labeling, p = phosphor protein, t = total protein.

Since cell proliferation was affected in most EGFR mutated cells, we hypothesized that *FAM83H-AS1* affects tumor progression through the EGFR pathway. We first performed receptor tyrosine kinase phosphorylation antibody array analysis, which included 49 different phosphorylated proteins covering most cancer-related pathways. We found that MET was the most affected protein after *FAM83H-AS1* knockdown by siRNA in the EGFR mutated cell PC-9 (Fig. 5A). We next performed Western blot to confirm this results in PC-9 and H1650 cells and found that EGFR, AKT and ERK1/2 were also affected after *FAM83H-AS1* knockdown (Fig. 5B–D). As indicated in Fig. 5, EGFR, MET and their downstream proteins AKT and ERK1/2 were significantly decreased after *FAM83H-AS1* knockdown in both PC-9 and H1650 cells. Identification of the *FAM83H-AS1* direct binding proteins using RNA pull-down assay or identification of the functional domains of *FAM83H-AS1* using domain-specific chromatin isolation by RNA purification (dChIRP)^30^ are currently being investigated.

In order to explore the gene expression pathways regulated by *FAM83H-AS1*, we performed RT-PCR on 20 genes which are involved in pathways of MET/EGFR, cell cycle, EMT, etc. We found MET mRNA was decreased by 40%. Other genes were not affected after *FAM83H-AS1* siRNA treatment on PC-9 cell (Supplementary Figure 6), indicating MET may be regulated at the transcription level and the others at the translational level.

## Discussion

Genome-wide analyses indicate that lncRNA expression can confer clinical information about patient outcome and may have utility in diagnostic tests^22^,^31^,^32^,^33^. For example, *MALAT1* not only has a crucial significance in predicting metastasis risk, but could also be a diagnostic biomarker detectable in the blood screening for lung cancer^34^,^35^. Prostate cancer gene 3 (*PCA3*) has been used as a biomarker in clinical practice for predicting prostate cancer volume^36^. *H19* expression was found to be higher in gastric cancer (GC) tissues^37^, and highly expressed in the plasma of patients with GC^38^. *HULC* is reportedly detectable in peripheral blood cells of HCC patients^39^. Patients with high expression of *HOTAIR* are more likely to have tumor recurrence and poor prognosis^40^. *HOTAIR* might also be a biomarker for lymph node metastasis in HCC^41^. The characterization of the RNA species, their functions, and their clinical application has therefore become an area of biological and clinical importance in cancer research. In our previous study, we identified lncRNAs differently expressed in lung cancer as compared to normal lung using three large RNA-Seq data sets. Some of the lncRNAs were also related to patient outcome in lung cancer. These lncRNAs could potentially serve as diagnostic or prognostic markers in lung cancer. In this study, we have validated the differently expressed lncRNA, *FAM83H-AS1*, in an independent set of LUADs using RT-PCR and found that *FAM83H-AS1* was significantly increased in cancer and correlated with worse patient survival. We also found that *FAM83H-AS1* was increased in squamous cell and large cell lung cancer, as well as bladder, breast, gastric, head/neck, and prostate cancers. Increased *FAM83H-AS1* expression in many types of cancers supports the hypothesis that this lncRNA plays a role in cancer progression and has potential use as a cancer biomarker. We found that knockdown of *FAM83H-AS1* by siRNA impairs tumor cell proliferation, migration and invasion. These effects may be through cell cycle regulation by inducing G2 arrest.

LncRNAs may represent good candidates as therapeutic targets^42^,^43^. *HOTAIR* can reduce the sensitivity of lung adenocarcinoma cells to chemotherapeutic drugs such as cisplatin^43^. Down-regulation of *MALAT1* expression reduced tumor growth *in vivo*^35^. EGFR is a major therapeutic target in EGFR mutated lung cancers. So far, all anti-EGFR therapies are targeted towards inhibition of its kinase activity, either by using small molecule tyrosine kinase inhibitors (erlotinib and gefitinib) or by monoclonal antibodies (cetuximab)^44^. However, clinical responses to such therapies have improved the outcome for only a subset of patients. Furthermore, erlotinib-sensitive patients often develop resistance due to acquisition of a T790M EGFR mutation^45^. Thus, there is an urgent need to identify additional strategies capable of targeting mutant EGFR. In this study, we found that MET/EGFR and their downstream signaling ERK1/2 and AKT may be the targets of *FAM83H-AS1* in lung cancer, indicating that *FAM83H-AS1* may be a potential therapeutic target. Further characterization of the *FAM83H-AS1* direct binding molecules is ongoing.

Gene expression profiling after *FAM83H-AS1* knockdown would provide more unbiased information regarding pathways affected at the transcriptional levels. In this study, we have only measured 20 genes involved in MET/EGFR, cell cycle, EMT, and other pathways. We found *MET* mRNA was decreased by 40%, but other genes were not affected after *FAM83H-AS1* knockdown indicating MET may be regulated at the transcriptional level and others at the translational levels.

We did not find a significant correlation between *FAM83H-AS1* expression and Ki-67 staining (Supplementary Figure 5). The cell growth in some cells (H358, H441 and HCC827) were not affected by *FAM83H-AS1* siRNA knockdown, although these cells have relatively higher levels of *FAM83H-AS1* expression, indicating these cells grow independent of *FAM83H-AS1* levels. Cell growth in other cells (H838, H1299 and H1975) were affected by *FAM83H-AS1* siRNA knockdown, although these cells have relatively low levels of *FAM83H-AS1* expression, indicating that cell growth is affected if *FAM83H-AS1* expression reaches a certain level (Supplementary Figure 3). This could partially explain why the correlation between *FAM83H-AS1* expression and Ki-67 expression was not significant. Another reason may be that Ki-67 also represents a subset of tumors with higher proliferation rate. This was supported by proliferation-related gene cluster analysis (Supplementary Figure 7). Finally, some other unknown genomic events may be involved.

*FAM83H-AS1* is a novel lncRNA with little known about its roles in primary tumors. Interestingly, using DAVID Gene Ontology analysis, we uncovered that *FAM83H-AS1* may be involved in the inhibition of immune response in lung cancer (data not shown). Further validation and characterization of this mechanism may support a role for this lncRNA in tumor immune regulation.

In summary, this study provides a comprehensive analysis of newly characterized lncRNA *FAM83H-AS1* in lung cancer. Future experiments including RNA pull-down, gene overexpression, and gene profiling affected by *FAM83H-AS1* are warranted in order to establish the roles of *FAM83H-AS1* in cancer progression and its potential use as a diagnostic or prognostic marker. Our findings also warrant further exploration of this lncRNA as a potential therapeutic target for patients with cancer.

## Methods

### Lung cancer/normal specimen

For qRT-PCR validation, 101 lung cancer and 19 paired non-tumor lung tissues were obtained from patients undergoing cancer surgery during the period from 1991 to 2012 at the University of Michigan Health System. All the patients provided informed consent, and all experimental protocols were approved by the University of Michigan Institutional Review Board and Ethics Committee. The methods were carried out in accordance with approved guidelines. Resected specimens were stored at −80 °C until use. Frozen tissues for regions containing a minimum of 70% tumor cellularity, defined by cryostat sectioning, were used for RNA isolation. None of the patients received any preoperative radiation or chemotherapy. The clinical information is shown in Supplementary Table 1. The median follow-up time was 8.1 years among the patients that remained alive.

### Lung cancer cell lines

All 12 lung cancer cell lines used in this study were obtained from the American Type Culture Collection and maintained in RPMI 1640, supplemented with 10% FBS and 1% antibiotic-antimycotic. Cells were cultured at 37 °C in a 5% CO2 cell culture incubator. Genotyping for identity of all cell lines was performed at the University of Michigan Sequencing Core, and testing of mycoplasma contamination was routinely examined.

### RNA isolation and quantitative RT-PCR

Total RNA isolation, cDNA synthesis, and qRT-PCR from tissue samples and cell lines were performed as described previously^12^. The oligonucleotide primers for *FAM83H-AS1* were forward: 5′-TAGGAAACGAGCGAGCCC, and reverse: 5′-GCTTTGGGTCTCCCCTTCTT. The housekeeping genes *GAPDH* and *ACTB* were used as loading controls. Fold-changes were calculated relative to control siRNA or the median value of normal tissue samples.

### Western Blot analysis

Lung cancer cell lysates were boiled in sample buffer, separated by polyacrylamide gel electrophoresis, and transferred onto polyvinylidene difluoride membranes. After blocking for 1 h with 5% non-fat milk or BSA, the membranes were incubated with primary antibodies against human MET (1:1000 dilution), EGFR (1:1000 dilution), AKT (1:1000 dilution), ERK1/2 (1:1000 dilution), and GAPDH (1:10,000 dilution) overnight at 4 °C. After incubation with HRP-conjugated secondary antibody (at a 1:10,000 dilution) for 1 h at room temperature, the membranes were developed using ECL and exposed to X-ray film.

### siRNA knockdown assay

Cells were plated at a desired cell number and 24 h after plating, target siRNAs and non-targeting controls (scribe siRNA #1 Dharmacon, USA) were transfected at a final concentration of 10 nM. Lipofectamine RNAiMax Reagent and OptiMEM medium were used for knockdown experiments according to the manufacturer’s instructions (Invitrogen, USA). SMARTpool of *FAM83-AS1* siRNA (Dharmacon) was used in this study. Knockdown efficiency was measured by qRT-PCR.

### Cell proliferation, invasion and migration assays

Detailed experiments of these assays were described previously^12^. In short, for cell proliferation, cells were plated in 96-well plates at a desired concentration and transfected *FAM83H-AS1* siRNA or non-targeting controls at 24 h after plating. At 96 h or 120 h after transfection with siRNAs, the proliferation rates were measured by Cell Proliferation Reagent (WST-1) (Roche) according to manufacturer’s instructions. For invasion and migration assays, cells were treated with the indicated siRNAs. After 48 h siRNA transfection, cells were seeded onto Boyden chambers (8 mm pore size, BD) present in the insert of a 24-well culture plate (Matrigel was used for invasion, purchased from BD). After 48 h, invasive cells located on the lower side of the chamber were stained with Diff-QuikTM Stain Set (SIEMENS).

### Flow cytometry for cell cycle analysis

PC-9 and H1650 cells were collected after 48 h treatment with *FAM83-AS1* siRNA and control siRNA. The cells were fixed with 70% ice-cold ethanol and washed with PBS. Cells were re-suspended in 1 ml of propidium iodide (PI) staining solution ((0.1% (v/v) Triton X-100, 10 μg/mL PI, and 100 μg/mL DNase-free RNase A in PBS), and then incubated for 30 minutes at room temperature in the dark. Cell cycle was measured in the University of Michigan flow cytometry core.

### Statistical analysis

Data were analyzed using GraphPad Prism 6 (GraphPad software) and R software. The Receiver Operating Characteristic (ROC) curve analysis was used for diagnostic accuracy measured by the area under the curve (AUC). The Kaplan-Meier Survival curves and the log-rank test were used for survival analysis. Other data, such as proliferation, invasion, and migration, were evaluated by unpaired Student’s t-test. A two-tailed p value < 0.05 was considered significant. The correlation of proliferation related genes was analyzed with hierarchical cluster and heat map was visualized using Tree View software. To determine potential underlying biological processes associated with *FAM83H-AS1* correlated (Pearson correlation) or regulated genes, Gene Ontology enrichment analysis was performed based on significantly correlated genes using the DAVID bioinformatics website^46^.

## Additional Information

**How to cite this article**: Zhang, J. *et al*. Overexpression of *FAM83H-AS1* indicates poor patient survival and knockdown impairs cell proliferation and invasion via MET/EGFR signaling in lung cancer. *Sci. Rep.* **7**, 42819; doi: 10.1038/srep42819 (2017).

**Publisher's note:** Springer Nature remains neutral with regard to jurisdictional claims in published maps and institutional affiliations.

## Supplementary Material

Supplementary Information
