# Supplementary material for: Overexpression of FAM83H-AS1 indicates poor patient survival and knockdown impairs cell proliferation and invasion via MET/EGFR signaling in lung cancer
Source: Sci Rep. 2017 Feb 15;7:42819. doi: 10.1038/srep42819 (PMC5309882; doi:10.1038/srep42819)
Supplement: Supplementary Information [file srep42819-s1.pdf]

Overexpression of *FAM83H-AS1* indicates poor patient survival and knockdown impairs cell proliferation and invasion via MET/EGFR signaling in lung cancer

Jie Zhang[1,2], Shumei Feng[3], Wenmei Su[4], Shengbin Bai[3], Lei Xiao[3], Lihui Wang[5], Dafydd G. Thomas[6], Jules Lin[2], Rishindra M. Reddy[2], Philip W. Carrott, Jr.[2], William R. Lynch[2], Andrew C. Chang[2], David G. Beer[2], You-min Guo[1], Guoan Chen[2]

[1]Xian Jiaotong University, Xi’an, China; [2]Department of Surgery, University of Michigan, Ann Arbor, Michigan, USA; [3]Xinjiang Medical University, Urumqi, China; [4]Guangdong Medical University, Zhanjiang, China; [5]Guangxi Medical University, Nanning, China; [6]Department of Pathology, University of Michigan

Supplementary data
2 Tables
7 figures

Supplementary Table 1. *FAM83H-AS1* expression and clinical variables from validation set of lung ADs measured by qRT-PCR

Variables	AD	FAM83H-AS1	*P* value*
Age			
<=65	42	1.34	
>65	59	1.7	0.6
Gender			
Female	53(52.5%)	1.58	
Male	48	1.64	0.6
Stage			
Stage I	59(58.4%)	1.19	
Stage II	16	2.1	0.4
Stage III	26	1.69	0.8
N status			
No	70	1.48	
N1-2	31	1.89	0.8
Differentiation			
Well	28	1.44	
Moderate	38	1.65	0.5
Poor	34(33.7%)	1.48	0.5

*t test was used.

Supplementary Table 2. *EGFR*, *KRAS* and *TP53* genes mutation in lung cancer cell lines used in Figure 3B

Cell Line	Subtype	EGFR	KRAS	TP53
PC-9	AD	E746_A750del	WT	WT
Hcc4006	AD	Del L747-E749	WT	WT
H1650	AD	E746_A750del	WT	WT
H838	AD	WT	WT, amp?	E62stop
H1975	AD	L858R, T790M	WT	WT
H1299	AD	WT	WT	trancated
H727	carcinoid	?	?	?
H358	AD	WT	G12C	WT
H441	AD	WT	G12V	R158L
SK-MES-1	NSCLC	WT	WT	E298stop
H2228	AD	WT	WT	Q331stop
HCC827	AD	Del E746-A750, EGFR amp	WT	WT

Note: H2228 cell line has *EML4-ALK* fusion gene; Amp: DNA amplification; del: deletion.

Supplementary Figure 1

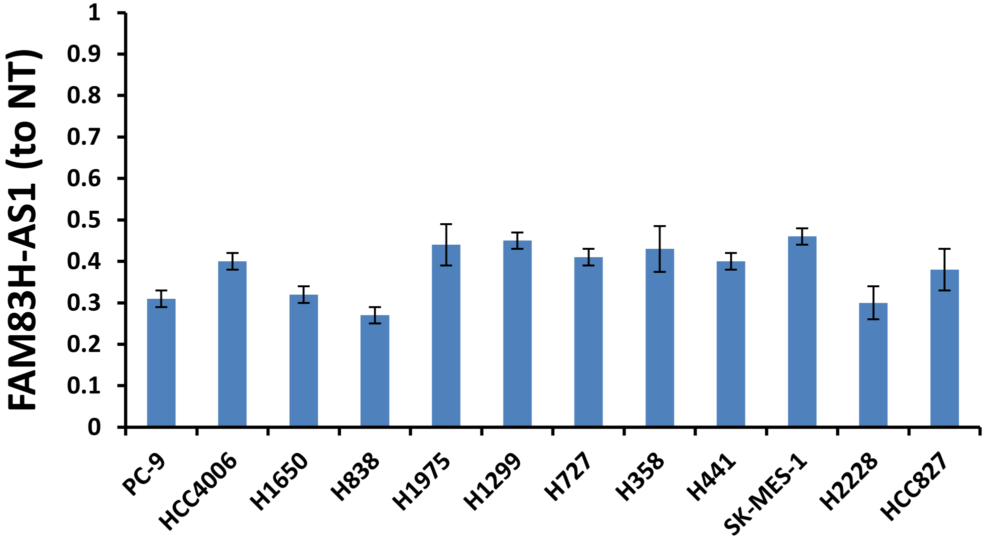

The knockdown efficiency of FAM83H-AS1 siRNA smartpool (10nM, 48 hrs) on 12 lung cancer cell lines used in this study on Figure 3B. (ACTB as mRNA loading control, all values are relative to NT control, NT=1)

Supplementary Figure 2

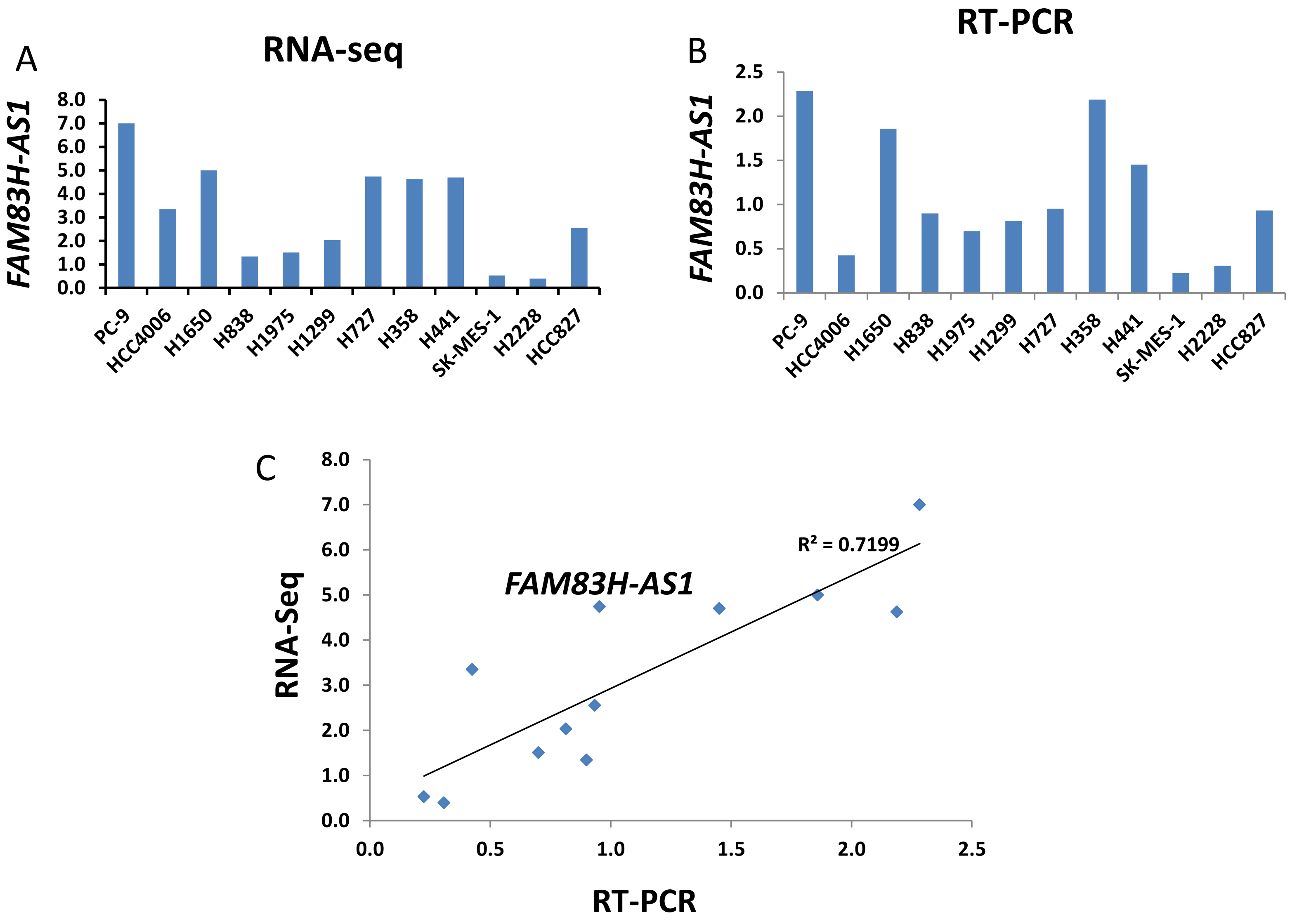

FAM83H-AS1 expression in 12 lung cancer cell lines used in this study showing in Figure 3B. **A**, RNA-Seq data showing *FAM83H-AS1* expression (log2 of FPKM value); **B**, RT-PCR validation of *FAM83H-AS1* expression (relative to median value of 12 cells); **C**, correlation between RT-PCR and RNA-Seq values in these 12 cell lines ($r = 0.72$, $p = 0.004$).

Supplementary Figure 3

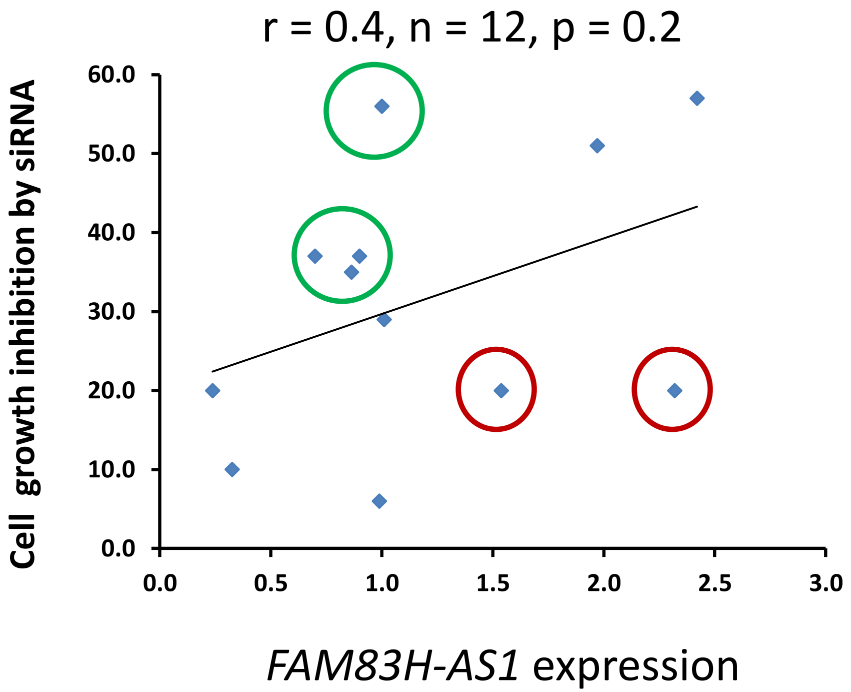

The correlation between *FAM83H-AS1* expression and rate of cell growth inhibition by *FAM83H-AS1* knockdown using siRNA. The cell growth in some cells (indicated by red cycle) were not affected by *FAM83H-AS1* siRNA knockdown although these cells have relative higher level of *FAM83H-AS1* expression. Whereas, cell growth in some cells (H838, H1299 and H1975) were affected by *FAM83H-AS1* siRNA knockdown although these cells have relative low level of *FAM83H-AS1* expression.

Supplementary Figure 4

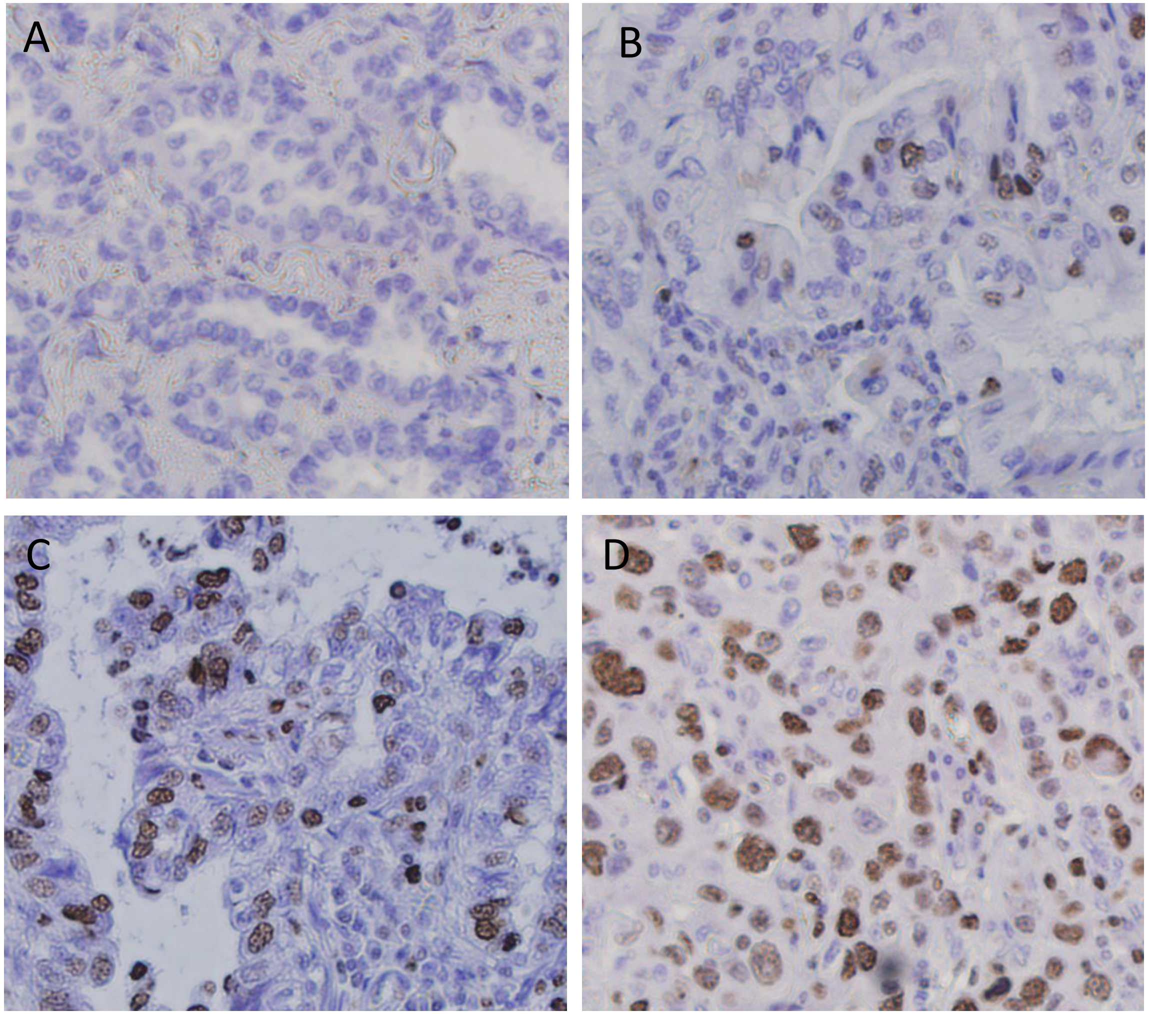

Ki-67 immunohistochemistry (IHC) staining on 97 lung cancer tissue array. Ki-67 staining was scored as 0 (negative, A), 1 (<10% positive cells, B), 2 (10-50% positive cells, C), or 3 (>50% positive cells, D).

Supplementary Figure 5

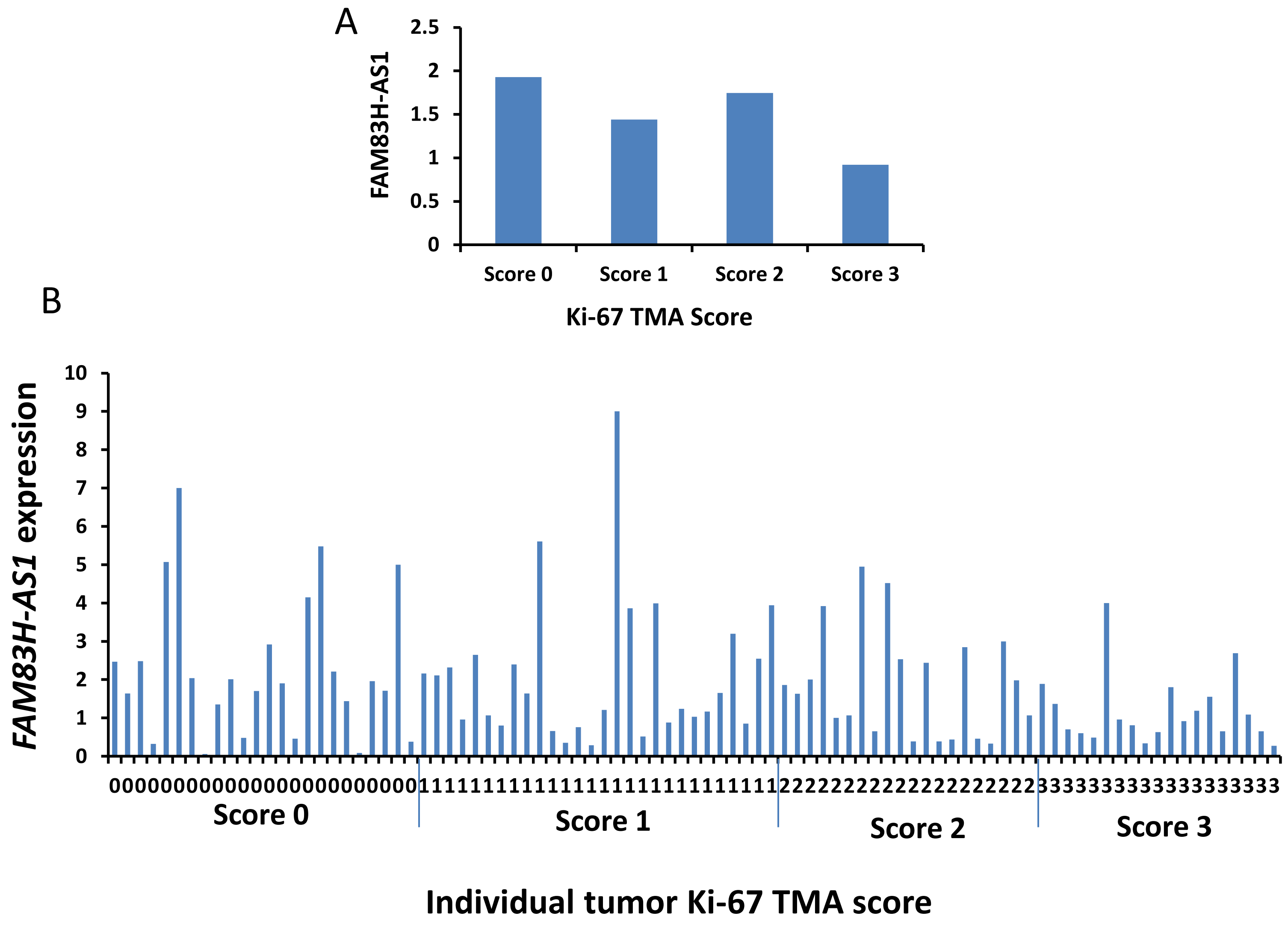

A, Median values of *FAM83H-AS1* expression in each Ki-67 score group; **B**, *FAM83H-AS1* expression and Ki-67 tissue array (TMA) score in each individual tumor sample.

Supplementary Figure 6

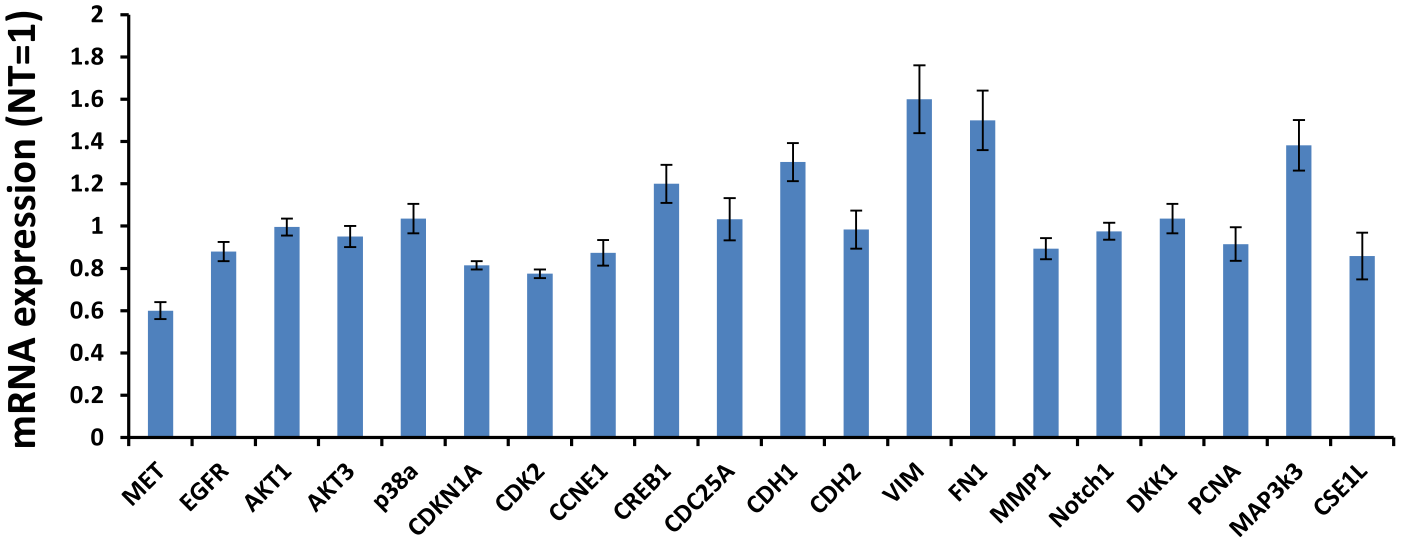

RT-PCR indicated the change in gene (mRNAs) expression after *FAM83H-AS1* siRNA (10nM) treatment at 48 hrs on PC-9 cell. MET mRNA was decreased by 40%, other genes were not affected. (ACTB as mRNA loading control, all values are relative to NT control).

Supplementary Figure 7

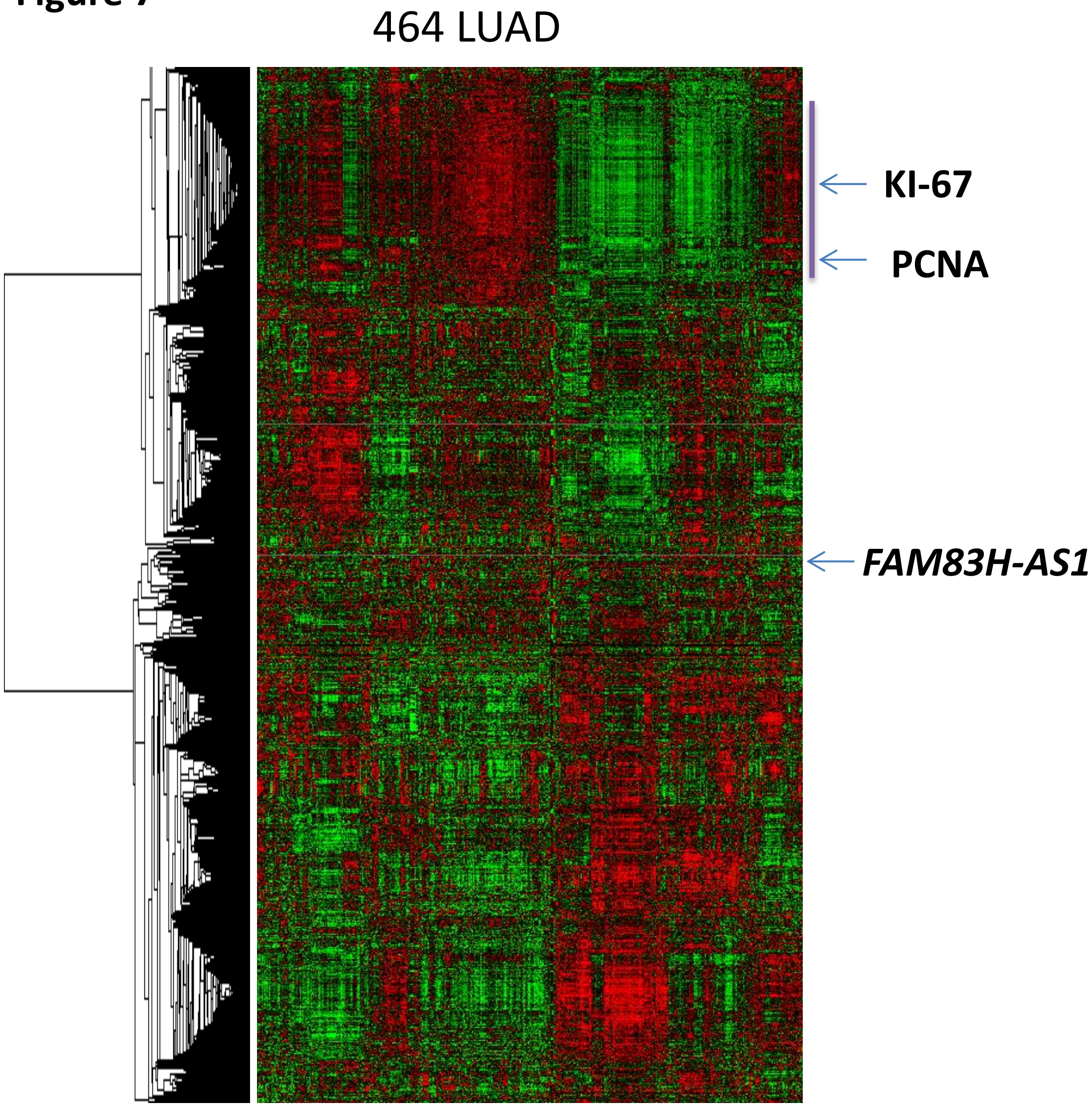

Heat maps of cluster analysis showing 868 proliferation related genes (downloaded from Ben-Porath, Nat. Gent. 2008) together with *FAM83H-AS1* from 464 lung ADs (UM 67, Seo 85 and TCGA 312) RNA-Seq data. Columns represent samples, and rows genes, red is high expression and green low expression. Heat map showing that about 2/3 LUADs have a higher Ki-67 expression and other 1/3 tumors were presented by other proliferation genes. Regarding the proliferation related genes, KI-67 only represented a small subset of these proliferation related genes. Most of the proliferation related genes are not cluster with Ki-67 or PCNA.
